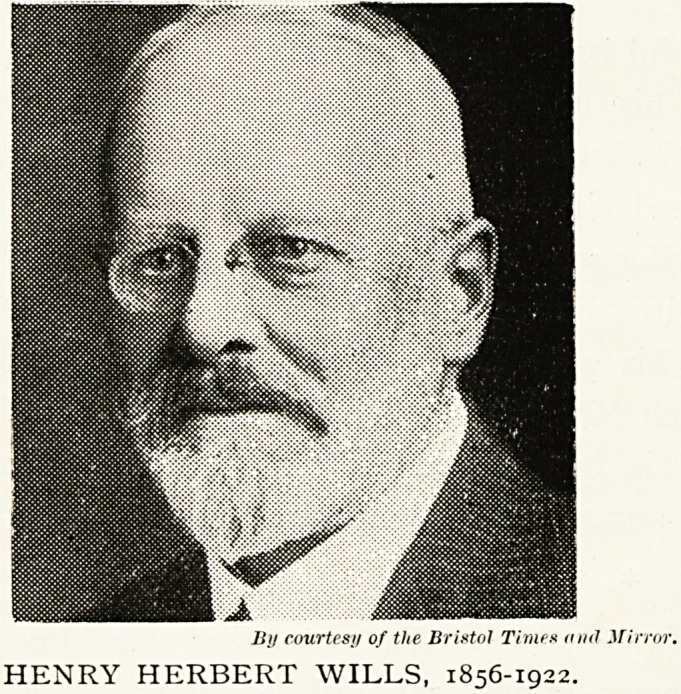# Editorial Notes

**Published:** 1922-06

**Authors:** 


### Editorial Notes.

**The Routine of Operations for Tonsils and Adenoids.**

UNDER the heading we borrow from the *British Medical Journal* (April 1st, 1922) attention is directed to the recommendations issued by the Council of the Laryngological Section of the Royal Society of Medicine on the hygienic conditions which should be observed in performing operations for the removal of tonsils and adenoids in hospitals and other clinics for children. The need for adequate experience in diseases of the ear and throat by the surgeon in charge of these cases is rightly emphasised, for ability to operate on tonsils and adenoids is not sufficient, what is equally important is diagnostic skill to recognise the various conditions which predispose to nasal catarrh and to differentiate between those cases where operations are absolutely necessary and those for whom other treatment is more suitable. Other points which are emphasised are (1) that all patients requiring operation for tonsils and adenoids ought to be kept in the hospital for at least forty-eight hours, and (2) that before they are discharged inquiry should be made as to the home conditions, especially with reference to the presence of infectious diseases, and (3) that printed instructions should be issued to the parents for the preparation of the patient before admission and also for after-treatment when the patient is discharged from the in-patient wards. But as it is impossible to treat all cases as in-patients until more adequate accommodation is available, the Council urge (4) that, when a child is to be operated on and sent home the same day, investigation should have always been made beforehand as to the adequacy of the home accommodation and supervision, and also the absence of adverse sanitary conditions. Further (5) that the child should be visited by a nurse after discharge, particularly in the case of children who are denied the safeguards of in-patient accommodation. And lastly (6) that every child who is sent home on the day of operation should be transferred in an ambulance or hired conveyance, and not left to the discretion of a poor parent to take it home in a public bus, tram or train.

We feel that these recommendations ought not to be necessary, and yet it is common knowledge that even these essentials have not been observed uniformly. To take a child from a dirty home, so crowded that it is impossible to keep the patient separated from other children, then without any inquiry as to the presence in the house of diphtheria or scarlatina, etc., to operate and send the child straight back to take its chance, is discreditable. Moreover, the recommendations of the Council as to the proper provision of adequate waiting-room accommodation, and that the recovery room should be scrupulously clean and well ventilated and provided with separate beds or couches for each patient, should be acted on by those institutions where such conditions do not already obtain. No institution should be allowed to continue to take the little children for operation on tonsils and adenoids or any other operation unless they can provide at least the essentials for their comfort and safety.

The Ministry of Health through its Educational Authorities have done an immense service in inspecting school children and in recommending the proper treatment to remove defects ; and further in many large towns and centres they have provided school clinics for this treatment to be carried out. For some diseases such provision has been made by the Bristol Education Committee, but we hold that, where such clinics are not provided, the Education Authorities should see to it that means for the carrying out of their recommendations are rendered available, either at voluntary hospitals, or failing that at the rate-supported hospitals. If the means at the disposal of the hospitals are inadequate, they should be supplemented by grants from the Educational Authority, subject of course to these institutions affording the essential special surgical service and accommodation. This would be true economy, for it is wasting public funds to provide medical inspectors to tell parents what treatment is required for their children without ensuring that such treatment is available within the limits of those parents' resources.

**The late Mr. H. H. Wills, Pro-Chancellor of the University of Bristol.**

WHILE this issue was in the press we learnt with profound regret that Mr. Harry Wills had died. Such a generous philanthropist, who has afforded such splendid service in promoting the Faculty of Medicine in Bristol, will ever be remembered in its University.

His public services and gifts took many directions, but we propose only to touch on Mr. Wills' work in developing medical work in our midst.

Perhaps the most striking movement that he initiated was the amalgamation scheme to fuse all the voluntary hospitals working under one general committee of direction so that economy in working, the development of large, highly-equipped departments and a united medical staff might result. The amalgamation scheme was not brought to completion, nevertheless it gave a new impetus to the work carried out at the voluntary hospitals. While avoiding either advocacy or criticism of that scheme, which may or may not fructify in future in some modified and perhaps improved form, the enthusiasm displayed by the late

Harry Wills has left its mark and merits our grateful remembrance. When this scheme was brought forward Mr. H. H. Wills was the President of the Royal Infirmary, his elder brother, Mr. George A. Wills, being President of the Bristol General Hospital. Before resigning his Presidency of the Royal Infirmary, owing to failing health, Mr. Wills gave £100,000 to the funds of that hospital. At the same time he continued to develop another project, by the purchase of Cote House at Westbury-on-Trym, where he proceeded to

*By courtesy of the Bristol Times and Mirror.*

HENRY HERBERT WILLS, 1856-1922.

build a Home for Incurables, St. Monica's Home, and he further set apart securities representing a sum of about one million pounds for the endowment of that institution.

Yet it is probable that even more far-reaching in its practical influence on medical work is the munificence of Mr. H. H. Wills in connection with the building of the University, and his gift of the new Physical Laboratory, to be built and equipped on the Royal Fort site that he had conveyed to the University. These, with many other noble gifts to his fellows to which we make no reference here, afford lasting evidence to future generations of Mr. Wills' wise generosity. But for those who enjoyed his personal acquaintance it is what he was, rather than all he has done, that leaves the deep sense of bereavement, since they alone knew the retiring, kindly disposition and warm heart of the man whose chief interest in life was to devise the most useful methods of spending and being spent for others. One of his characteristics was to throw himself heart and soul into the work and schemes he initiated, and he devoted an almost meticulous care to details, so as to ensure perfectly successful issues to his efforts.

To his brother, Mr. George A. Wills, with whom he joined in building the new University buildings now so far advanced, it must be a sore grief that his fellow Pro-Chancellor will not be with us to see the completion of their joint gift, the magnificent University buildings.

To his widow, whose collaboration in the many works to which Mr. Wills set his hand, was so invaluable, as well as to his brothers and other members of his family, we tender our sincerest sympathy.

**Professor James Swain, C.B., C.B.E., retires from practice.**

We note that Dr. James Swain has relinquished practice as a consulting surgeon, an announcement that will cause widespread regret to all his colleagues and professional confrères. To retire at the zenith of a great reputation, full of honours, yet with many years of unfettered activity for other interests, is an achievement many would envy and few could emulate.

We are glad to know that he will continue in his Clifton residence, and that his exploration of fresh fields will not take him from our midst.

## Figures and Tables

**Figure f1:**